# Correlation of human sperm intracellular pH in non-normozoospermic men with fertilization rates in assisted reproduction procedures

**DOI:** 10.1007/s10815-025-03736-7

**Published:** 2025-11-04

**Authors:** Paulina Torres-Rodríguez, Gabriela Carrasquel-Martínez, Arturo Matamoros Volante, Andrés Aragón-Martínez, Diana Lisbeth Flores, Israel Maldonado, Claudia L. Treviño

**Affiliations:** 1 Instituto de Biotecnología, Universidad Nacional Autónoma de México, Cuernavaca, Morelos Mexico; 2 Centro de Innovación Tecnológica y Medicina Reproductiva, CITMER, Mexico City, Mexico; 3 School of Biomedical and Chemical Engineering, Colorado State University, Fort Collins, CO 80523 USA; 4 Carrera de Biología, Laboratorio de Gametos y Desarrollo Tecnológico, Facultad de Estudios Superiores Iztacala, Unidad de Biomedicina, Universidad Nacional Autónoma de México, Tlalnepantla, Estado de México Mexico

**Keywords:** Human sperm pH_i_, ICSI, IVF, Fertilization rate, ART

## Abstract

**Purpose:**

Intracellular pH (pH_i_) in sperm cells plays a crucial role in various physiological processes, including motility, capacitation, and fertilization. While previous studies have shown a positive correlation between sperm pH_i_ and fertilization success in normozoospermic patients undergoing fertility treatments, its role in non-normozoospermic individuals is unclear.

**Methods:**

This study investigates the relationship between sperm pH_i_ and fertilization outcomes in patients undergoing assisted reproduction techniques: in vitro fertilization (IVF) and intracytoplasmic sperm injection (ICSI). Qualitative sperm pH_i_ evaluation was performed using time-lapse flow cytometry, and both basal pH_i_ and pH_i_ response capacity (delta pH_i_) were assessed in sperm samples from patients diagnosed with teratozoospermia, asthenoteratozoospermia, or hypoteratozoospermia.

**Results:**

Our results revealed significant differences in pH_i_ values among diagnostic groups and specific correlation patterns according to the ART used. In ICSI cycles, higher basal pHi values and reduced delta pH_i_ were significantly associated with higher fertilization rates in patients with teratozoospermia, while in IVF, the correlations were more variable.

**Conclusions:**

These findings suggest that measuring sperm pH_i_ could potentially serve as a valuable tool for predicting fertilization success and guiding treatment decisions during assisted reproduction techniques (ART), contributing to a better understanding of the molecular mechanisms underlying male infertility.

## Introduction

Human infertility is a medical condition characterized by the inability of a couple to conceive a pregnancy after 12 months of regular, unprotected sexual intercourse and affects 17.5% of adults globally [1]. Among male factors, sperm dysfunctions are considered the most frequent etiology of fertility problems. Male factors, including genetic, lifestyle, and unknown issues, account for approximately half of all infertility cases [1]. Current protocols for sperm analysis do not successfully predict the fertilizing capacity of semen samples from men seeking reproductive treatment. The most common approach to detecting such dysfunctions in sperm is through the evaluation of macro- and microscopic semen parameters, commonly referred to as semen analysis or seminogram. Individuals whose values fall within the reference ranges are classified as normozoospermic and are presumed to be fertile. However, the utility of these parameters as predictors of reproductive outcomes has been a topic of long-standing debate. Interestingly, the latest edition of the WHO manual for semen handling (2021) now recommends the development of techniques for functional sperm analysis, which may offer greater predictive value for fertility [2].

Sperm cells precisely adjust their pH_i_ to maintain a dynamic balance between production, elimination, transport, and buffering of H^+^ within cells at specific times, helping to regulate different functions [3, 4]. Several key sperm proteins are regulated by pH_i_, including the Ca^2+^ channel CatSper (sperm cation channel), which is involved in sperm hyperactivation [5, 6] and the K^+^ selective ion channel SLO3, which participates in membrane potential regulation during capacitation [7, 8]. When sperm encounter a medium that induces capacitation, the pH_i_ increases in the head and principal piece of the flagellum with different kinetics [9]. Thus, sperm possess the ability to regulate pH_i_ in a spatial–temporal manner [3, 9, 10]. The final stages of fertilization involve complex membrane interactions between sperm and egg, and changes in sperm pH_i_ may influence this process.

One study showed that sperm pH_i_ from normozoospermic infertile patients positively correlates with both hyperactivated motility and conventional in vitro fertilization (IVF) success [11]. Additionally, our group reported that normozoospermic men with a proven paternity history display a pH_i_ increase during in vitro capacitation, which is absent or less frequent in men of unproven paternity [9], demonstrating pH_i_’s relevance to sperm fertilizing capacity. To our knowledge, pH_i_ evaluations in non-normozoospermic patients had not been conducted. This is particularly significant since in our fertility clinic, approximately 95% of the semen samples present different grades of teratozoospermia diagnosis. Teratozoospermia is defined as a semen sample with less than 4% of morphologically normal sperm according to the WHO manual [2]. The majority of the samples in the present study presented isolated teratozoospermia, but some also presented asthenoteratozoospermia (reduced motility and normal morphology) or hypoteratozoospermia (reduced volume and normal morphology). Several studies have demonstrated a correlation between sperm morphological abnormalities and other affectations in the sperm such as nuclear genetic defects, apoptotic alterations, and increased reactive oxidative stress, all of which can adversely affect fertility potential [12–14]. Consequently, intracytoplasmic sperm injection (ICSI) is frequently recommended for men with teratozoospermia. However, the existing literature presents inconsistent findings regarding the impact of teratozoospermia on pregnancy outcomes and the success of ART proceedings [15, 16]. Notably, one study reported that isolated teratozoospermia was more prevalent among fertile males compared to infertile males [17]. For these reasons, pH_i_ evaluation in capacitated sperm from semen samples with teratozoospermia diagnosis of men undergoing ART treatment could serve as a valuable tool to help clinicians select optimal fertilization techniques for each couple.

Multicenter randomized controlled studies have been conducted to compare the efficacy of ICSI and conventional IVF in couples with non-severe male infertility, aiming to provide valuable insights for optimal treatment selection in such cases. The choice between ICSI and IVF involves multiple factors, including the severity of male infertility, semen quality, previous ART cycle outcomes, financial considerations, and the couple’s personal preferences [18]. In this context, identifying relevant sperm markers to inform decisions on the most effective ART approach is an expanding and important research field. This study assessed whether a correlation exists between sperm basal pH_i_ and pH_i_ response capacity (delta pH) with levels and fertilization rates, considering different seminal diagnoses in patients undergoing ART (IVF and ICSI), and further explored the potential use of sperm pH_i_ levels as a predictive marker of fertilizing capability for both techniques.

## Materials and methods

### Ethics statement and inclusion criteria

This study included semen samples from patients who attended the assisted reproduction clinic for in vitro fertilization (IVF) or intracytoplasmic sperm injection (ICSI) treatments between January 2023 and March 2025. All semen samples were subjected to a classical seminogram test, and the following parameters were determined manually: semen volume, sperm concentration (millions/mL), percentage of normal morphology, and percentage of progressive motility. Semen samples were classified following the protocols described in the WHO Laboratory Manual for the Examination and Processing of Human Semen [2] into three diagnostic groups: (1) teratozoospermia (T): percentage of spermatozoa with normal morphology below the reference value (< 4%), 129 semen samples; (2) asthenoteratozoospermia (AT): combination of reduced motility (< 32% of spermatozoa with progressive motility) and morphological alterations, 15 semen samples; and (3) hypoteratozoospermia (HT): combination of reduced semen volume (< 1.5 mL) and morphological abnormality, 19 semen samples. All samples were anonymized and handled following ethical standards approved by the fertilization clinic. Sperm samples in ART cycles involving four or more inseminated oocytes were included. Frozen semen samples were excluded. For this study, only the surplus of the seminal samples available after completing their respective ART procedures was employed. The results of this investigation did not influence technicians, clinicians, or patients’ treatment decisions. Given that this study is subject to a confidentiality agreement with the fertilization clinic, only fertilization rate data were available to assess ART success and were used to correlate with the pH_i_ levels and seminogram parameters determined for each sample.

To have pH_i_ reference values, this study also included semen samples from 13 men (donors) with a normal seminogram (sperm concentration × 10^6^/mL ≥ 16, progressive motility (%) ≥ 32, normal morphology (%) ≥ 4). These samples were not subjected to ART treatments. All participants (donors and patients) signed an informed consent for the use of their samples for research purposes.

### Medium and reagents

The capacitating medium used was HTF HEPES medium (Human Tubal Fluid medium; InVitroCare Inc., CA, USA), supplemented with 10% w/v human serum albumin (HSA; InVitroCare Inc., CA, USA). The fluorescent dye 2′,7′-Bis-(2-Carboxyethyl)−5-(and-6)-Carboxyfluorescein, Acetoxymethyl Ester (BCECF-AM) was sourced from Thermo Fisher Scientific (Waltham, MA, USA), and propidium iodide (PI) was obtained from Molecular Probes-Invitrogen, Inc. (Eugene, OR, USA). Stock solutions of fluorescent dyes and test compounds were prepared in dimethyl sulfoxide (DMSO), except for NH_4_Cl, which was prepared in tri-distilled water.

### Sperm sample preparation

Semen samples (from donors and patients) were collected by masturbation in sterile containers after sexual abstinence of 2–5 days. Since only patients’ samples were subjected to ART, these samples were collected on the same day as oocyte retrieval. After the sample liquefied for approximately 10–15 min at room temperature, motile spermatozoa were recovered using the density gradient technique according to WHO guidelines [2], with the upper (90%) and lower (45%) layers of SpermCare (InVitroCare Inc., CA, USA) gradient solutions. Briefly, solutions were layered sequentially in a 5 mL tube, arranged from the lowest to the highest concentration in a 1:1 v/v ratio, and finally an equal volume of the semen sample was placed at the top of the tube. The tube with the gradient was centrifuged at 1200 × *g* for 10 min at room temperature, and the pellet obtained was washed once with fresh pre-warmed HTF medium and centrifuged again at 1200 × *g* for 7 min at room temperature. The supernatant was discarded, and the pellet was resuspended in 70–160 µL of pre-warmed HTF medium to be used in further ART cycles. After the ART treatment was completed, the surplus sperm sample was adjusted to a concentration of 1 × 10^6^ sperm/mL using a Makler chamber (Sefi-Medical Instruments ltd. Haifa, Israel). This adjusted sperm suspension was used for flow cytometry analysis.

### Single-sperm selection andpH_i_ data acquisition

**Fig. 1:**
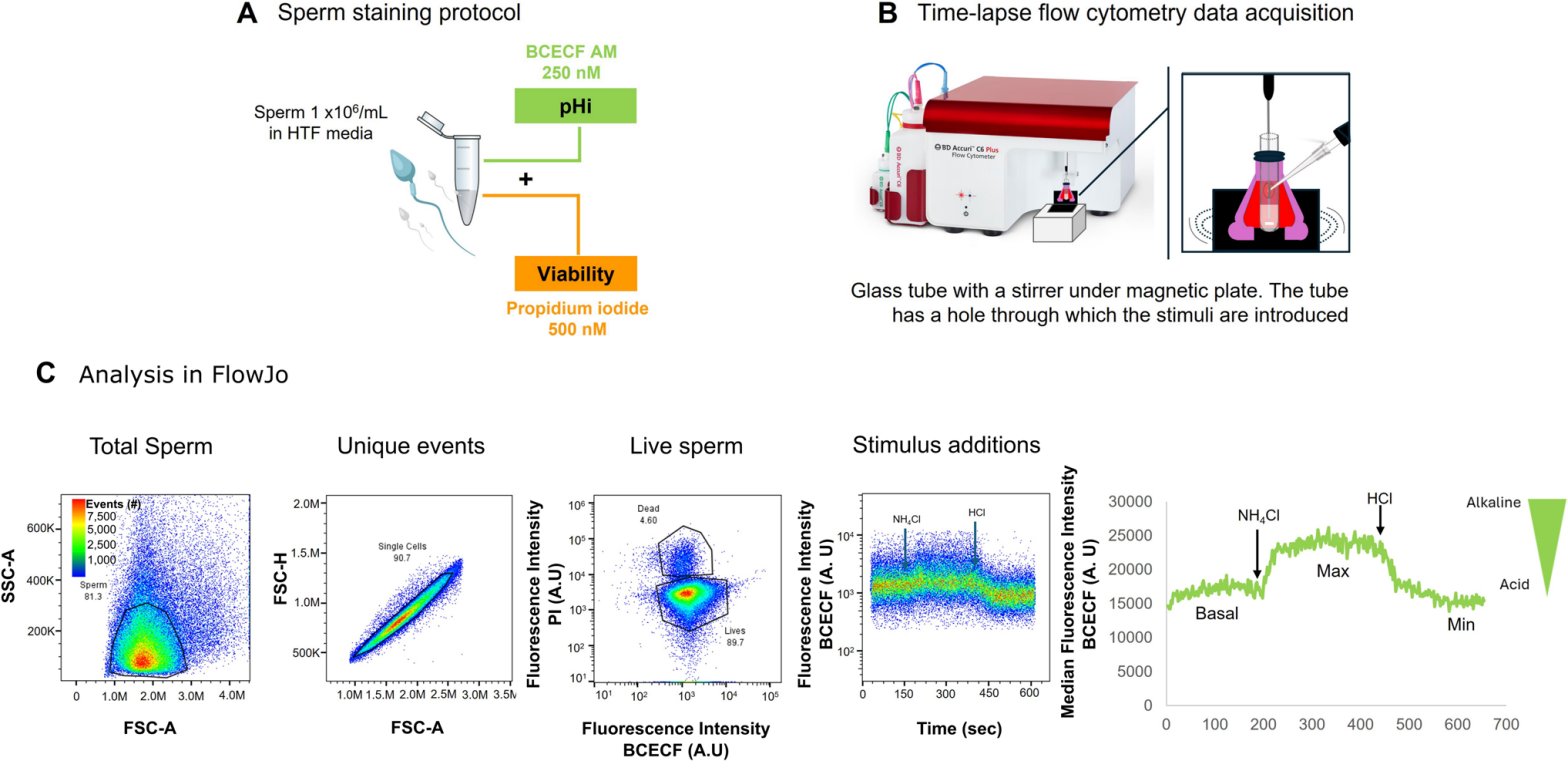
Human sperm pHi measurement protocol using a BD Accuri™ C6 Plus Flow Cytometer.** A** Diagram of sperm double staining with the pH_i_-sensitive dye BCECF-AM and propidium iodide (PI) as a viability marker. **B** Representation of the BD Accuri™ C6 Plus cytometer showing the adaptation to allow adding and mixing of different compounds with a micropipette into the tube during constant fluorescence recording. **C** Sequence of data analysis using FlowJo software, in order to finally select only single live sperm. Forward and side scatter properties (FSC and SSC) were recorded for each sample, and threshold values were established to eliminate debris and cell fragments. Subsequently, a two-dimensional density plot of FSC height and area was employed to isolate spermatozoa from cell aggregates. Only live (PI-negative) cells were subjected to further analysis. The addition of NH_4_Cl enables the recording of maximum fluorescence (due to alkalinization), which is then used to normalize the basal pH_i_ value (F/Fmax) and to calculate the delta pHi. The delta pHi represents the difference between the maximum response elicited by NH_4_Cl and the basal fluorescence level prior to stimulation (Fmax-F). As a control that the cells respond properly, HCl was added at the end of the trace to induce an acidification which produces a decrease in fluorescence. A representative trace showing the median fluorescence after each stimulus is shown

The fluorescent pH_i_-sensitive dye BCECF-AM was used to assess sperm pH_i_ qualitatively. Sperm were stained with 250 nM BCECF-AM for 10 min, and prior to data acquisition, 500 nM of the fluorescent viability marker, PI, was added to select only the population of live cells (Fig. 1A). Upon entering cells, BCECF-AM undergoes enzymatic hydrolysis of its acetoxymethyl ester (AM) group, resulting in the accumulation of free BCECF within the cytoplasm. BCECF fluorescence increases with alkaline pH_i_ and decreases in acidic environments.

Individual cellular events were captured using a BD Accuri C6 Plus flow cytometer (Becton Dickinson, Franklin Lakes, NJ, USA) (Fig. 1B). Measurements were conducted at a constant temperature (23 °C) in continuous mode, allowing for time-lapse analysis at a flow rate of 14 µL/min. BCECF fluorescence and PI were detected by exciting the sample with a 488-nm laser, and emission was collected using a 533/30-nm filter (FL-1 channel) for PI and a 670 LP filter (FL-3 channel) for BCECF fluorescence, respectively. Sperm were selected using FSC-A and SSC-A values; doublets and aggregates were discarded from the analysis by selecting only unique events through comparison of area (FSC-A) and height (FSC-H). Only viable individual cells were included in the analysis, and dead cells were excluded through PI staining (PI +) (Fig. 1C). The baseline BCECF fluorescence was recorded for the first 60 s (named basal pH_i_). Subsequently, 20 mM NH₄Cl was added to induce intracellular alkalinization (observed as a BCECF fluorescence increase). Finally, 5 mM HCl was added to induce intracellular acidification (observed as a BCECF fluorescence decrease). The total duration of the recordings was 10 min.

To further evaluate sperm BCECF fluorescence in a qualitative manner, the average BCECF fluorescence level (F) was normalized using the maximum response achieved by the addition of 20 mM NH₄Cl (named Fmax). This F/Fmax ratio was also employed to equalize the variability of BCECF fluorescence readings from different samples (Fig. 1C). Additionally, a delta pH_i_ value was calculated by subtracting the basal *F* value from the Fmax. This represents the difference between the maximum fluorescence reached after the addition of NH₄Cl and the basal pH_i_ fluorescence. As sperm become alkalinized, the delta pH_i_ decreases because the NH₄Cl alkalinization response is smaller. Conversely, the more acidic the basal pH_i_, the greater the delta pH_i_. The pH_i_ response capacity or delta pH_i_ is an indirect measure of sperm pH_i_ and its regulation.

### Fertilization rates for ART treatments

The decision to use ICSI or IVF was made and performed by expert clinical embryologists, independently of this study and not by the authors of the present work. Therefore, we did not include the detailed ART procedures, as they were not a direct part of this study. Of the total 163 ART cycles analyzed during this study, the basal pH_i_ levels of sperm from 67 samples were assigned to patients undergoing ICSI, while 96 samples were allocated to patients inseminated via conventional IVF protocols. The outcomes of those treatments were provided to us to correlate with our pH_i_ measurements. The fertilization rate was calculated by embryologists by dividing the number of zygotes by the total number of mature oocytes [19]. Additionally, samples from 13 donors were evaluated to provide a reference of basal pH_i_ values from healthy men. Sperm samples from donors were not subjected to ART treatment.

### Statistical analysis

Flow cytometry experimental data from the BD Accuri C6 Plus were saved in the standard FCS format. The FCS files were first analyzed using FlowJo Software version 10.1 by FlowJo, LLC (Ashland, USA). Cytometry data and values of seminal parameters were used to construct a dataset in comma-separated values (CSV) format for further analysis. Differences in fluorescence values were analyzed using one-way ANOVA with a model of orthogonal contrasts (linear contrasts). Correlations among values of fluorescence variables, fertilization rate, and parameters from seminogram were analyzed by Pearson correlation test. For all tests, *p* < 0.05 was considered significant.

All statistical analyses were performed in R version 4.4.1 [20] and Python version 3.12.2 running in Jupyter Notebook version 7.2.2 using Anaconda version 2.6.3. Plots were constructed with ggplot2 version 3.5.1 [21] and matplotlib version 3.9.2. Final figures were produced using Inkscape 0.91 (Inkscape.org).

## Results

### Comparison of semen parameters and basal pH_i_ between normozoospermic and non-normozoospermic men

We first compared the basic parameters of semen analysis between our reference group of normozoospermic donors (*n* = 13; NORMO) and our study group of non-normozoospermic patients (*n* = 163; NON-NORMO). Semen samples from the patients were classified as teratozoospermic (*n* = 129; T), asthenoteratozoospermic (*n* = 15; AT), and hypoteratozoospermic (*n* = 19; HT) according to the 2010 WHO Manual [2]. The average values for each group are shown in Table 1. Since the difference in semen volume, motility, and morphology is used to classify the samples into each diagnostic category, we could only compare the sperm concentration among samples. The average values (expressed as millions of cells per mL of semen) were 108 for HT, 42 for AT, 91 for T, and 93 for NORMO. Interestingly, there were statistically significant differences among the samples: AT vs. HT *p* = 0.0002, AT vs. NORMO *p* = 0.002, and AT vs. T *p* = 0.0001 (using Kruskal–Wallis with Dunn’s post-hoc test) (Table 1).

**Table 1:** List of patients and donors processed. This table displays data from 163 patients and 13 donors, detailing their clinical profiles, assisted reproductive technology (ART) outcomes, pH basal, and delta pH

#	Oocytes protocol	Semen volume (mL)	Concentration (cells × 10^6)	Morphology (%)	Motility (%)	Diagnostic	Basal pHi	Delta pHi	Female age (years)	Male age (years)	Obtained oocytes (number)	ART procedure	Assigned IVF	Assigned ICSI	Zygote total	Fertilization	Fertilization rate (%)
1	Patient	2.6	57	1	52	T	0.819	1.315	43	45	9	IVF	9	0	8	0.89	89
2	Patient	3.5	112	1	58	T	0.871	1.153	37	32	15	IVF	15	0	12	0.80	80
3	Patient	3.5	112	1	58	T	0.815	1.226	37	32	14	IVF	14	0	6	0.43	43
4	Patient	6	106	1	44	T	0.742	1.385	38	38	7	IVF	7	0	3	0.43	43
5	Patient	2.3	79	2	48	T	0.781	1.291	39	38	20	ICSI	0	13	13	1.00	100
6	Patient	1.3	145	1	65	HT	0.751	1.314	36	38	7	IVF	7	0	2	0.29	29
7	Patient	4	34	0	53	T	0.872	1.180	43	43	8	ICSI	0	6	3	0.50	50
8	Patient	1.5	126	1	56	T	0.774	1.321	43	42	10	ICSI	0	9	7	0.78	78
9	Patient	1.5	70	2	60	T	0.850	1.179	39	33	7	IVF	7	0	4	0.57	57
10	Patient	5.8	64	2	62	T	0.860	1.163	41	29	4	IVF	4	0	2	0.50	50
11	Patient	1.5	126	1	56	T	0.792	1.282	43	42	10	ICSI	0	9	7	0.78	78
12	Patient	1.5	126	1	56	T	0.882	1.145	43	42	10	ICSI	0	9	7	0.78	78
13	Patient	2.4	81	2	54	T	0.861	1.170	40	40	15	IVF	15	0	15	1.00	100
14	Patient	3.5	65	0	55	T	0.813	1.230	40	41	20	ICSI	0	20	17	0.85	85
15	Patient	2.1	61	0	55	T	0.877	1.166	49	42	9	ICSI	0	8	7	0.88	88
16	Patient	2.1	61	0	55	T	0.869	1.148	49	42	8	ICSI	0	7	4	0.57	57
17	Patient	2.6	92	1	60	T	0.851	1.204	43	40	13	IVF	13	0	12	0.92	92
17	Patient	2.6	92	1	60	T	0.851	1.204	43	40	12	ICSI	0	11	10	0.91	91
18	Patient	1.1	153	2	58	HT	0.861	1.152	35	39	9	IVF	9	0	6	0.67	67
19	Patient	2.5	63	0		T	0.729	1.379	36	36	14	ICSI	0	11	11	1.00	100
20	Patient	2.5	111	1	56	T	0.866	1.131	37	48	7	IVF	7	0	3	0.43	43
21	Patient	2.5	111	1	56	T	0.892	1.097	37	48	4	ICSI	0	4	2	0.50	50
22	Patient	2.8	80	1	54	T	0.936	1.107	45	36	20	IVF	20	0	15	0.75	75
23	Patient	1.7	220	1	61	T	0.934	1.076	37	37	12	ICSI	0	7	7	1.00	100
24	Patient	1.9	129	2	63	T	0.881	1.159	32	35	4	IVF	4	0	1	0.25	25
25	Patient	2	120	1	48	T	0.883	1.137	42	35	10	IVF	10	0	9	0.90	90
26	Patient	2.1	72	1	50	T	0.899	1.126	27	28	23	IVF	12	0	7	0.58	58
27	Patient	2.1	72	1	50	T	0.899	1.126	27	28	23	ICSI	0	7	7	1.00	100
28	Patient	5	35	0	55	T	0.866	1.154	41	43	14	ICSI	0	14	12	0.86	86
29	Patient	1	52	1	50	HT	0.813	1.247	39	40	13	ICSI	0	10	7	0.70	70
30	Patient	5.8	58	1	53	T	0.795	1.233	38	43	4	IVF	4	4	0	0.00	0
31	Patient	5.8	58	1	53	T	0.795	1.233	38	43	4	ICSI	0	4	4	1.00	100
32	Patient	1.6	167	2	54	T	0.795	1.257	40	40	7	IVF	7	0	2	0.29	29
33	Patient	4.2	136	2	69	T	0.903	1.116	34	36	8	ICSI	0	8	6	0.75	75
34	Patient	3	50	1	32	T	0.820	1.224	43	34	12	IVF	12	0	8	0.67	67
35	Patient	1.7	52	0	53	T	0.674	1.407	36	35	22	ICSI	0	22	13	0.59	59
36	Patient	1.9	8.2	0	34	T	0.703	1.483	32	36	18	ICSI	0	14	5	0.36	36
37	Patient	1.8	96	2	51	T	0.664	1.584	38	43	11	IVF	11	0	8	0.73	73
38	Patient	1.8	96	2	51	T	0.664	1.584	38	43	10	ICSI	0	10	8	0.80	80
39	Patient	3.1	30	1	35	T	0.728	1.415	28	32	7	IVF	6	0	5	0.83	83
40	Patient	3.1	30	1	35	T	0.728	1.415	28	32	7	ICSI	0	6	6	1.00	100
41	Patient	3.1	140	3	57	T	0.860	1.170	30	31	5	IVF	5	0	3	0.60	60
42	Patient	4.1	92	2	60	T	0.812	1.234	39	41	7	IVF	7	0	3	0.43	43
43	Patient	2.3	150	1	70	T	0.714	1.359	28	38	17	IVF	17	0	1	0.06	6
44	Patient	2.6	54	1	35	T	0.747	1.254	38	36	10	IVF	10	0	8	0.80	80
45	Patient	2.6	54	1	35	T	0.747	1.254	38	36	10	ICSI	0	10	2	0.20	20
46	Patient	1.7	150	1	60	T	0.746	1.304	39	54	9	IVF	9	0	3	0.33	33
47	Patient	2.3	108	2	50	T	0.748	1.339	34	38	10	IVF	10	0	4	0.40	40
48	Patient	5.3	63	1	55	T	0.685	1.462	34	38	15	IVF	15	0	4	0.27	27
49	Patient	1.3	133	1	37	HT	0.739	1.367	41	39	12	IVF	12	0	10	0.83	83
50	Patient	1.3	133	1	37	HT	0.739	1.367	41	39	12	ICSI	0	11	8	0.73	73
51	Patient	3	53	1	55	T	0.653	1.534	42	36	6	IVF	6	0	5	0.83	83
52	Patient	1.2	110	0	60	HT	0.717	1.416	40	38	10	ICSI	0	8	6	0.75	75
53	Patient	2.5	119	1	62	T	0.732	1.390	35	32	10	IVF	10	0	3	0.30	30
54	Patient	2.1	145	1	53	T	0.711	1.417	42	43	5	IVF	5	0	4	0.80	80
55	Patient	2.1	145	1	53	T	0.711	1.417	42	43	6	ICSI	0	6	5	0.83	83
56	Patient	3	66	1	25	AT	0.754	1.350	42	40	20	ICSI	0	13	8	0.62	62
57	Patient	4.7	118	1	39	T	0.757	1.348	32	39	11	IVF	11	0	9	0.82	82
58	Patient	2.3	46	0	32	T	0.710	1.362	36	44	5	ICSI	0	5	3	0.60	60
59	Patient	3.5	150	1	61	T	0.702	1.339	39	41	10	ICSI	0	10	6	0.60	60
60	Patient	2.1	69	1	60	T	0.719	1.392	29	39	10	IVF	10	0	4	0.40	40
61	Patient	2.1	69	1	60	T	0.719	1.392	29	39	10	ICSI	0	8	5	0.63	63
62	Patient	3.1	170	1	59	T	0.770	1.343	40	44	6	IVF	6	0	2	0.33	33
63	Patient	1.7	120	1	76	T	0.857	1.189	41	41	8	ICSI	0	7	5	0.71	71
64	Patient	7	140	1	51	T	0.734	1.393	39	43	21	IVF	21	0	18	0.86	86
65	Patient	7	140	1	51	T	0.709	1.443	39	43	20	ICSI	0	18	8	0.44	44
66	Patient	2.4	138	2	58	T	0.758	1.335	45	47	25	IVF	25	0	12	0.48	48
67	Patient	1.6	133	2	68	T	0.857	1.167	41	39	8	IVF	8	0	6	0.75	75
68	Patient		128	2	49	T	0.804	1.248	36	39	15	IVF	11	0	9	0.82	82
69	Patient	2.8	104	1	62	T	0.745	1.339	39	36	10	IVF	10	0	10	1.00	100
70	Patient	1.2	320	2	66	HT	0.818	1.218	41	41	8	IVF	7	0	5	0.71	71
71	Patient	2.8	59	0	50	T	0.667	1.566	41	43	4	ICSI	0	4	2	0.50	50
72	Patient	4.7	70	1	64	T	0.618	1.607	37	35	5	IVF	5	0	2	0.40	40
73	Patient	3.4	58	1	21	AT	1.039	0.989	40	36	16	ICSI	0	13	10	0.77	77
74	Patient	1.6	75	2	49	T	1.028	0.983	36	51	17	IVF	17	0	12	0.71	71
75	Patient	1.5	36	1	58	T	0.983	1.022	39	32	4	IVF	4	0	3	0.75	75
76	Patient	1.4	76	2	45	HT	0.791	1.294	41	38	8	IVF	8	0	5	0.63	63
77	Patient	1.2	37	0	27	HT	0.772	1.257	37	39	8	ICSI	0	8	6	0.75	75
78	Patient	1.5	121	3	55	HT	0.763	1.308	41	37	4	IVF	4	0	2	0.50	50
79	Patient	1	80	1	32	HT	0.783	1.281	37	36	16	ICSI	0	10	6	0.60	60
80	Patient	1.3	158	2	48	HT	0.624	1.607	35	44	7	IVF	7	0	5	0.71	71
81	Patient	1.4	49	0	58	HT	0.721	1.396	40	40	10	ICSI	0	7	7	1.00	100
82	Patient	1	85	1	58	HT	0.888	1.150	42	39	4	IVF	4	0	1	0.25	25
83	Patient	4	3.9	0	53	T	0.731	1.359	39	43	12	ICSI	0	8	7	0.88	88
84	Patient	4.2	26	1	29	AT	0.863	1.118	32	37	7	ICSI	0	6	4	0.67	67
85	Patient	2	30	0	56	T	0.936	1.114	45	33	8	IVF	8	0	6	0.75	75
86	Patient	3.3	31	0	24	AT	0.857	1.173	34	34	5	ICSI	0	4	1	0.25	25
87	Patient	2	43	1	28	AT	0.836	1.217	33	40	4	ICSI	0	3	1	0.33	33
88	Patient	2	43	1	28	AT	0.901	1.309	33	40	4	ICSI	0	3	1	0.33	33
89	Patient	1.1	105	1	41	HT	0.803	1.248	35	42	8	IVF	8	0	7	0.88	88
90	Patient	1.1	105	1	41	HT	0.721	1.365	35	42	9	IVF	9	0	7	0.78	78
91	Patient	2	145	2	54	T	0.742	1.372	36	39	6	IVF	6	0	4	0.67	67
92	Patient	2	34	1	19	AT	0.845	1.168	47	47	51	ICSI	0	45	40	0.89	89
93	Patient	1.5	185	1	66	T	0.748	1.337	35	35	9	IVF	9	0	5	0.56	56
94	Patient	1.5	185	1	66	T	0.738	1.326	35	35	8	IVF	8	0	5	0.63	63
95	Patient	2.4	41	1	44	T	0.770	1.291	41	34	7	ICSI	0	7	6	0.86	86
96	Patient	2.4	41	1	44	T	0.733	1.359	41	34	8	IVF	8	0	4	0.50	50
97	Patient	2.1	158	1	57	T	0.675	1.487	42	41	4	ICSI	0	3	0	0.00	0
98	Patient	3.7	68	1	31	AT	0.906	1.149	28	30	5	IVF	5	0	4	0.80	80
99	Patient	3.7	68	1	31	AT	0.906	1.149	28	30	5	ICSI	0	5	2	0.40	40
100	Patient	3	46	1	39	T	0.803	1.226	21	20	8	IVF	8	0	5	0.63	63
101	Patient	2.4	56	1	30	AT	0.895	1.118	30	44	11	IVF	11	0	9	0.82	82
102	Patient	2.4	56	1	30	AT	0.993	1.035	30	44	11	ICSI	0	11	8	0.73	73
103	Patient	5	30	2	59	T	0.886	1.112	38	38	20	ICSI	0	13	7	0.54	54
104	Patient	2.6	34	1	56	T	0.823	1.180	30	36	8	ICSI	0	8	8	1.00	100
105	Patient	2.3	32	1	53	T	0.862	1.171	39	41	4	IVF	4	0	2	0.50	50
106	Patient	5	128	2	49	T	0.813	1.241	36	39	15	IVF	11	0	9	0.82	82
107	Patient	1.2	95	1	38	HT	0.678	1.464	37	41	32	ICSI	0	25	20	0.80	80
108	Patient	1.8	6.3	0	23	AT	0.707	1.412	36	41	26	ICSI	0	20	17	0.85	85
109	Patient	1.1	48	1	53	HT	0.780	1.341	43	41	6	ICSI	0	6	5	0.83	83
110	Patient	2.2	228	2	72	T	0.738	1.381	41	40	5	IVF	5	0	5	1.00	100
111	Patient	2	57	1	64	T	0.886	1.175	40	36	6	ICSI	0	5	4	0.80	80
112	Patient	2.3	55	2	35	T	0.913	1.103	29	30	6	ICSI	0	5	4	0.80	80
113	Patient	2.3	55	2	35	T	0.845	1.212	29	30	6	ICSI	0	6	6	1.00	100
114	Patient	2.4	58	3	55	T	0.759	1.337	37	32	4	IVF	4	0	4	1.00	100
115	Patient	2.4	58	3	55	T	0.756	1.355	37	32	4	IVF	4	0	3	0.75	75
116	Patient	3	55	1	53	T	0.597	1.677	38	35	26	IVF	15	0	14	0.93	93
117	Patient	2	58	0	41	T	0.781	1.272	30	30	8	ICSI	0	7	4	0.57	57
118	Patient	4.8	35	2	63	T	0.930	1.075	33	33	4	IVF	4	0	4	1.00	100
119	Patient	4.8	35	2	63	T	0.865	1.213	33	33	4	IVF	4	0	4	1.00	100
120	Patient	7	140	1	51	T	0.734	1.393	39	43	21	IVF	21	0	18	0.86	86
121	Patient	7	140	1	51	T	0.709	1.443	39	43	20	ICSI	0	18	8	0.44	44
122	Patient	1.5	48	2	63	T	0.795	1.285	32	31	6	IVF	6	0	4	0.67	67
123	Patient	2.1	125	2	50	T	0.732	1.369	38	39	8	IVF	8	0	6	0.75	75
124	Patient	3.2	24	0	24	AT	0.742	1.441	41	39	15	ICSI	0	12	9	0.75	75
125	Patient	2.6	50	3	74	T	0.686	1.481	37	36	19	IVF	19	0	13	0.68	68
126	Patient	3.3	56	1	33	T	0.762	1.314	40	42	20	ICSI	0	19	15	0.79	79
127	Patient	3.2	150	2	63	T	0.767	1.302	38	34	11	IVF	11	0	10	0.91	91
128	Patient	3.2	150	2	63	T	0.751	1.333	38	34	14	IVF	14	0	10	0.71	71
129	Patient	1.6	133	2	68	T	0.784	1.266	41	39	4	IVF	4	0	3	0.75	75
130	Patient	1.6	133	2	68	T	0.836	1.208	41	39	4	IVF	4	0	3	0.75	75
131	Patient	3.2	91	0	47	T	0.828	1.180	30	31	10	ICSI	0	8	8	1.00	100
132	Patient	3.2	91	0	47	T	0.813	1.194	30	31	10	ICSI	0	8	4	0.50	50
133	Patient	2.8	104	1	62	T	0.737	1.354	39	36	10	IVF	10	0	10	1.00	100
134	Patient	2.8	60	1	58	T	0.717	1.417	39	33	20	IVF	20	0	7	0.35	35
135	Patient	4.1	43	1	46	T	0.718	1.407	26	32	16	IVF	8	0	4	0.50	50
136	Patient	2.3	150	1	70	T	0.714	1.359	28	38	17	IVF	17	0	1	0.06	6
137	Patient	2.6	54	1	35	T	0.747	1.254	38	36	10	IVF	10	0	8	0.80	80
138	Patient	2.6	54	1	35	T	0.747	1.254	38	36	10	ICSI	0	10	2	0.20	20
139	Patient	2.3	108	2	50	T	0.748	1.339	34	38	10	IVF	10	0	4	0.40	40
140	Patient	5.3	63	1	55	T	0.685	1.462	34	38	15	IVF	15	0	4	0.27	27
141	Patient	3	53	1	55	T	0.653	1.534	42	36	6	IVF	6	0	5	0.83	83
142	Patient	2.6	54	1	35	T	0.747	1.254	38	36	10	IVF	10	0	8	0.80	80
143	Patient	2.6	54	1	35	T	0.747	1.254	38	36	10	ICSI	0	10	2	0.20	20
144	Patient	3	53	1	55	T	0.653	1.534	42	36	6	IVF	6	0	5	0.83	83
145	Patient	2.3	108	2	50	T	0.748	1.339	34	38	10	IVF	10	0	4	0.40	40
146	Patient	5.3	63	1	55	T	0.685	1.462	34	38	15	IVF	15	0	4	0.27	27
147	Patient	4.1	92	2	60	T	0.812	1.234	39	41	7	IVF	7	0	3	0.43	43
148	Patient	1.3	133	1	37	HT	0.739	1.367	41	39	12	IVF	12	0	10	0.83	83
149	Patient	1.3	133	1	37	HT	0.739	1.367	41	39	12	ICSI	0	11	8	0.73	73
150	Patient	1.7	120	1	76	T	0.857	1.189	41	41	8	ICSI	0	7	5	0.71	71
151	Patient	2.4	138	2	58	T	0.758	1.335	45	47	25	IVF	25	0	12	0.48	48
152	Patient	7	140	1	51	T	0.734	1.393	39	43	20	ICSI	0	18	8	0.44	44
153	Patient	7	140	1	51	T	0.709	1.443	39	43	21	IVF	21	0	18	0.86	86
154	Patient	2.7	80	1	48	T	0.962	1.041	41	37	4	ICSI	4	0	3	0.75	75
155	Patient	2.7	80	1	48	T	0.777	1.292	41	37	4	IVF	0	4	3	0.75	75
156	Patient	2.7	50	0	44	T	0.718	1.414	37	43	6	IVF	6	0	4	0.67	67
157	Patient	1.2	24	1	45	HT	0.682	1.478	38	41	7	ICSI	0	3	2	0.67	67
158	Patient	5.5	135	1	71	T	0.726	1.361	36	41	7	IVF	7	0	4	0.57	57
159	Patient	5.5	135	1	71	T	0.984	1.017	36	41	6	IVF	6	0	3	0.50	50
160	Patient	2	66	2	48	T	0.683	1.506	43	41	4	IVF	4	0	1	0.25	25
161	Patient	2	66	2	48	T	0.764	1.366	43	41	5	IVF	5	0	4	0.80	80
162	Patient	3.8	25	3	23	AT	0.718	1.417	43	45	8	IVF	8	0	7	0.88	88
163	Patient	3.8	25	3	23	AT	0.718	1.417	43	45	14	ICSI	0	14	12	0.86	86
1	Donor	ND	64	4	59	Normozoospermic	0.933	1.071		24							
2	Donor	ND	210	4	78	Normozoospermic	0.901	1.042		46							
3	Donor	1.3	93	4	54	Normozoospermic	0.763	1.309		33							
4	Donor	9	130	4	69	Normozoospermic	0.707	1.406		31							
5	Donor	3.4	58	4	72	Normozoospermic	0.654	1.449		28							
6	Donor	2.1	87	4	59	Normozoospermic	0.608	1.646		27							
7	Donor	1.5	81	4	63	Normozoospermic	0.681	1.490		31							
8	Donor	2.6	71	4	52	Normozoospermic	0.638	1.578		20							
9	Donor	2.3	73	7	67	Normozoospermic	0.737	1.343		25							
10	Donor	3.2	78	4	52	Normozoospermic	0.740	1.390		28							
11	Donor	1.6	87	4	58	Normozoospermic	0.716	1.414		33							
12	Donor	3.6	96	4	73	Normozoospermic	0.724	1.299		19							
13	Donor	1.7	78	4	61	Normozoospermic	0.910	1.102		30							

^*^ND not determined

### Assessment of sperm basal pH_i_ by time-lapse flow cytometry

**Fig. 2:**
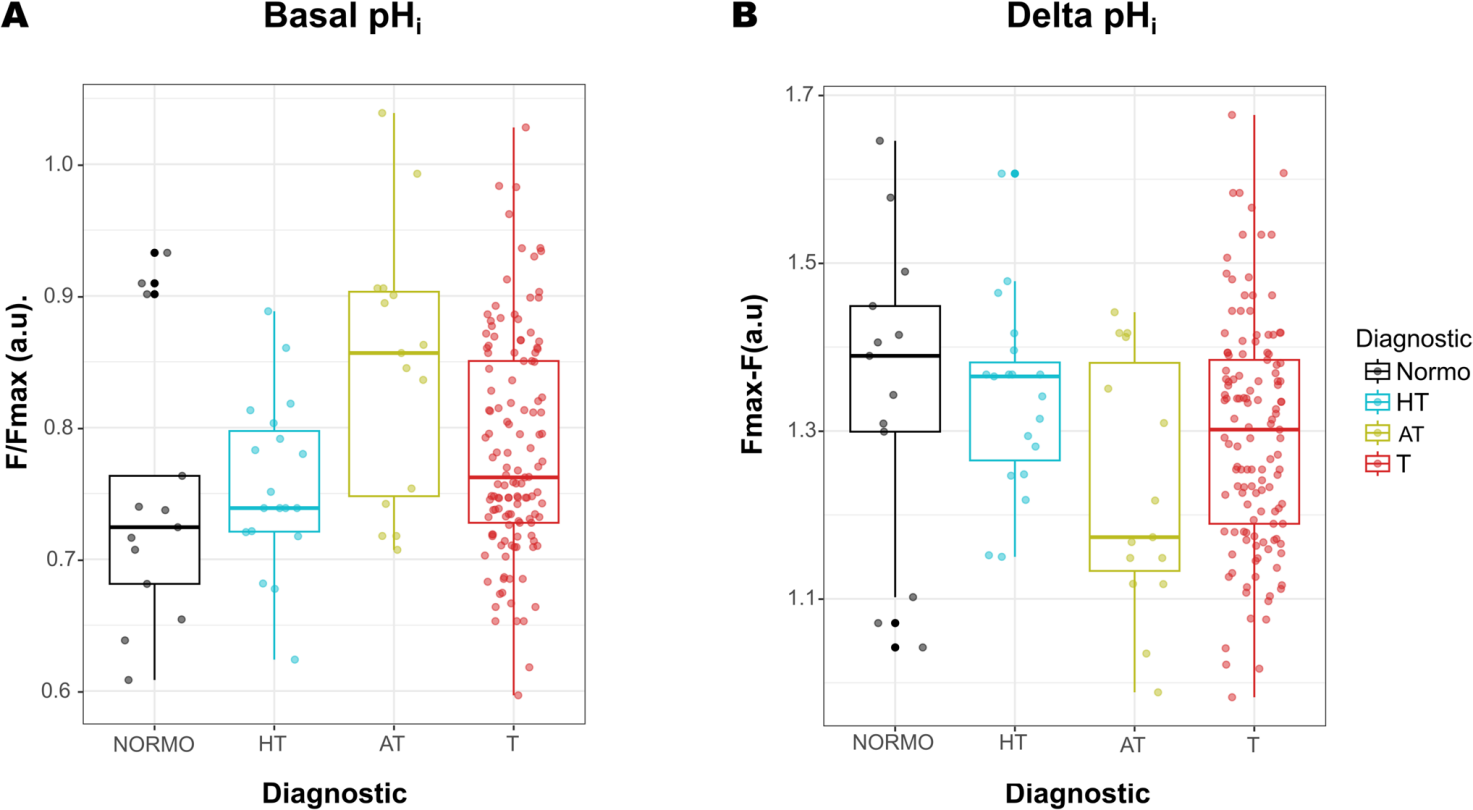
Determination of qualitative basal pH_i_values from non-normozoospermic men undergoing different ART treatments. Boxplots showing normalized BCECF fluorescence that represents basal pH_i_ (**A**) and Delta pH_i_ (**B**) values, comparing sperm samples from hypoteratozoospermic (HT), asthenoteratozoospermic (AT), and teratozoospermic (T) patients. Fluorescence values in non-normozoospermic groups were compared to those of the normozoospermic group. Comparisons were examined by one-way ANOVA analysis in a model of linear contrasts (orthogonal contrasts). Boxes represent the interquartile range (IQR), spanning from the first quartile (Q1) to the third quartile (Q3), with the median indicated by the horizontal line inside each box. Whiskers extend to the most extreme values within 1.5 × IQR (IQR = Q3 − Q1) below Q1 and above Q3. Data points outside this range are plotted as outliers. Colored dots are the data points measured in each group. Kruskal–Wallis with post-hoc (Dunn with Bonferroni test) analysis of data from (**A**) and (**B**) revealed that there were not significant differences among any of the groups

Subsequently, we qualitatively evaluated sperm basal pH_i_ levels utilizing the fluorescence of the pH-sensitive dye BCECF. We plotted the median basal pH_i_ (Fig. 2A) and delta pH_i_ (Fig. 2B) fluorescence values for each sperm sample. We found that all groups (AT, HT, and T) showed a more alkaline basal pH_i_ (i.e., higher fluorescence values) compared to normozoospermic controls; however, we only found significant differences in AT vs. N (*p* = 0.0005), and among the other groups, the differences were not statistically significant (Fig. 2A). Consistently, the delta pH_i_ values were lower in AT, HT, and T samples compared to normozoospermic controls; in this case, we detected a significant difference only for the AT group compared to the normozoospermic control (*p* = 0.009) (Fig. 2B).

**Fig. 3:**
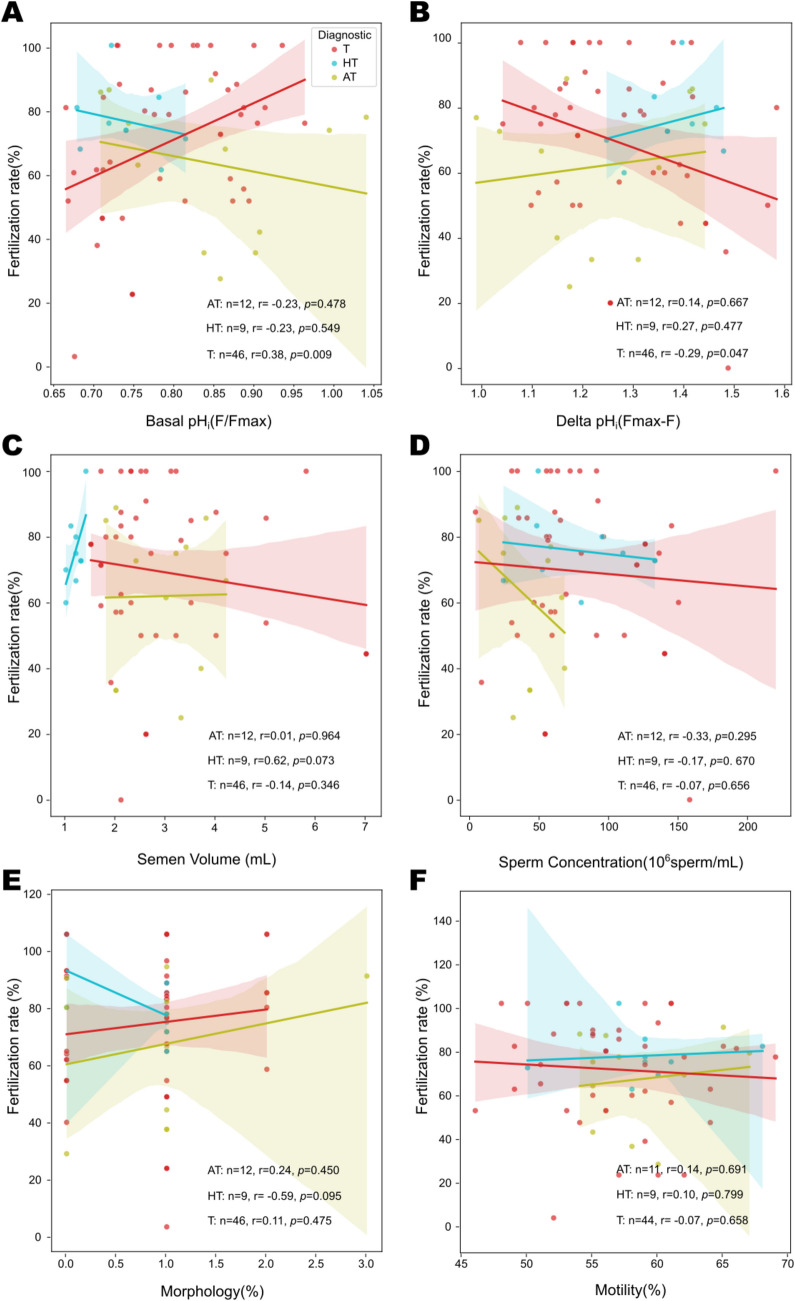
Sperm pH_i_alkalinization, but not semen parameters, correlates with fertilization rate success in ICSI. The percentage of fertilization rate was plotted against basal pHi (**A**), Delta pHi (**B**), semen volume (**C**), sperm concentration (**D**), percentage of normal morphology (**E**), and percentage of motility (**F**). The Pearson correlation *r* and *p* values are shown for each diagnostic group: teratozoospermic (T, red), hypoteratozoospermic (HT, blue), and asthenoteratozoospermic (AT, green). A significant correlation was found between fertilization rates only for pH_i_ values. The more alkaline the basal pHi and the smaller the Delta pHi, the higher the fertilization rate. The shaded area represents the 95% confidence interval

Additionally, we investigated whether the basal sperm pH_i_ differed between sperm samples in each group AT, HT, and T undergoing ICSI (*n* = 67) and those undergoing conventional IVF (*n* = 96). For the analysis, we performed Pearson’s coefficient analyses (*r* value) and evaluated the significance (*p* value). For ICSI treatment, we detected that only for the teratozoospermic group (marked in red), there was a correlation between the basal pH_i_ value and the fertilization rate. This correlation is positive (*r* = 0.38) although moderate, but highly significant (*p* = 0.009) (Fig. 3A). This indicates that the more alkaline the basal pH_i_, the higher the fertilization success rate in patients undergoing ICSI. Consistently, delta pHᵢ values were inversely correlated with fertilization rates, meaning that as delta pHᵢ value decreases, the fertilization rate increases (Fig. 3B). Delta pHᵢ values correlated significantly only in patients with teratozoospermia, a weak negative correlation with an *r* value of − 0.29, but significant (*p* = 0.047).

To underscore the importance of the correlation found with pHᵢ values, we examined whether there was any weighting of the fertilization rate with respect to the macroscopic parameters of the sample, namely semen volume (Fig. 3C), sperm concentration (Fig. 3D), percentage of normal morphology (Fig. 3E), and percentage of motile sperm (Fig. 3F). We found that there were non-significant correlations of these semen parameters with the fertilization rate.

**Fig. 4:**
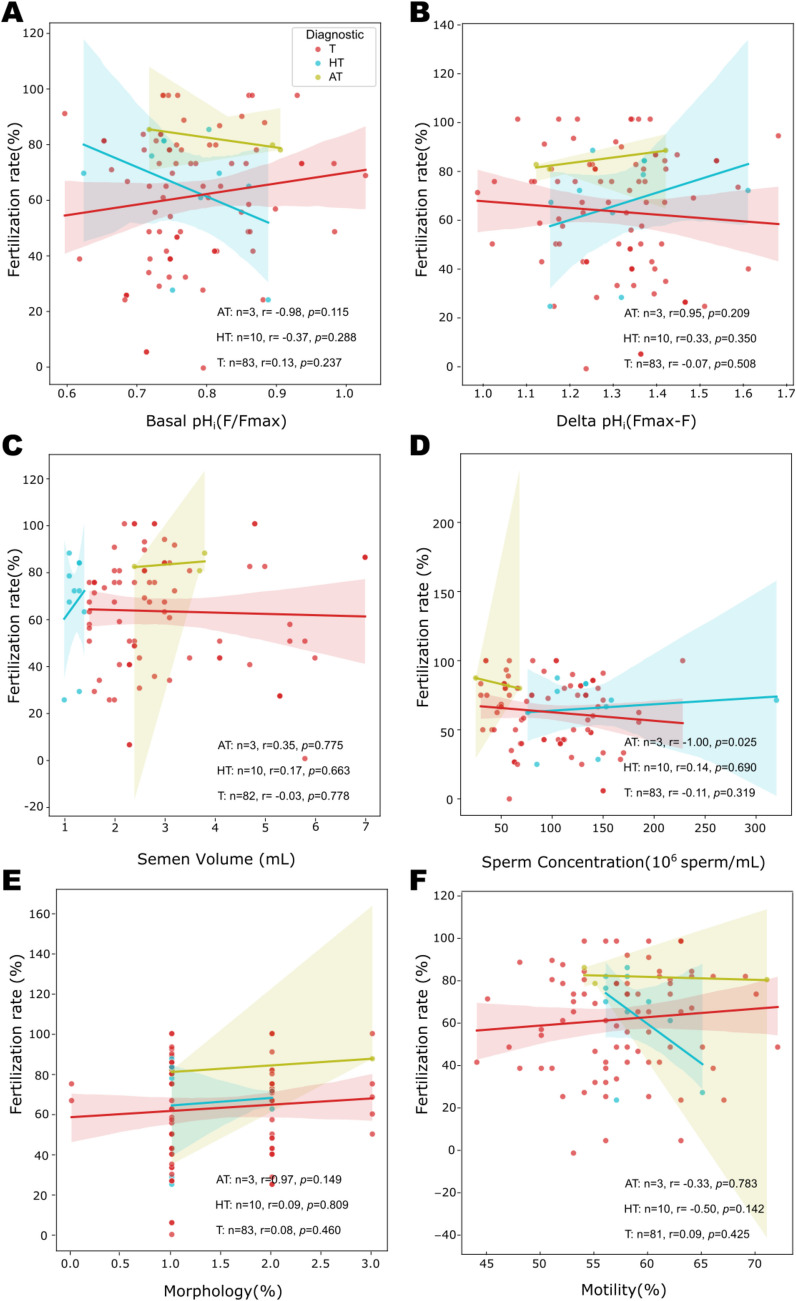
Sperm pHi and semen parameters do not correlate with fertilization rate success in IVF. The percentage of fertilization rate was plotted against basal pH_i_ (**A**), Delta pH_i_ (**B**), semen volume (**C**), sperm concentration (**D**), percentage of normal morphology (**E**), and percentage of motility (**F**). The Pearson correlation *r* and *p* values are shown for each diagnostic group: teratozoospermic (T, red), hypoteratozoospermic (HT, blue), and asthenoteratozoospermic (AT, green). The shaded area represents the 95% confidence interval. There is no significant correlation between fertilization rate and the parameters studied

Gunderson et al. [11] conducted a study showing that there is a positive correlation between higher sperm pHᵢ values and conventional IVF fertilization rates in sperm samples from normozoospermic patients. In our study, when we performed the Pearson correlation between fertilization rates and basal sperm pHᵢ or delta pHᵢ for each group of non-normozoospermic samples (AT, HT, and T) that underwent IVF, we did not find any correlation or significant differences, either for basal pHᵢ (Fig. 4A) or delta pHᵢ (Fig. 4B). The sperm samples had the following statistical values: AT (basal pHᵢ, *p* = 0.98; delta pHᵢ, *p* = 0.95), HT (basal pHᵢ, *p* = 0.28; delta pHᵢ, *p* = 0.29), and T (basal pHᵢ, *p* = 0.219; delta pHᵢ, *p* = 0.13).

For the macroscopic semen parameters, we detected a significant correlation between the fertilization rate and semen concentration for AT patients (marked in green), with a negative *r* = −1.00 value and a statistical significance of *p* = 0.025; however, this group is very small (*n* = 3), and further research is necessary to draw a conclusion (Fig. 4C). We did not find any significant correlation for the other semen parameters: sperm concentration (Fig. 4D), morphology (Fig. 4E), and motility (Fig. 4F) with fertilization rate.

Finally, Table 2 shows a summary of the data obtained from the correlations between fertilization rates and different variables evaluated in both ART procedures.

**Table 2:** Pearson correlation coefficients (*r*) and corresponding *p*-values for variables. This table presents the *r* and *p* values for the relationship between the fertilization rate and different variables studied in patients undergoing in vitro fertilization (IVF) or intracytoplasmic sperm injection (ICSI) treatments

ART procedure	X variable	Y variable	Diagnostic	*n*	Pearson *r*	*p*-value
ICSI	Basal pHi (F/Fmax)	Fertilization rate (%)	AT	12	–0.23	0.478
			HT	9	–0.23	0.549
			T	46	0.38	**0.009**
	Delta pHi		AT	12	0.14	0.667
			HT	9	0.27	0.477
			T	46	–0.29	**0.047**
	Semen volume (mL)		AT	12	0.01	0.964
			HT	9	0.62	0.073
			T	46	–0.14	0.346
	Concentration (cells × 10^6)		AT	12	–0.33	0.295
			HT	9	–0.17	0.67
			T	46	–0.07	0.656
	Morphology (%)		AT	12	0.24	0.45
			HT	9	–0.59	0.095
			T	46	0.11	0.476
	Motility (%)		AT	11	0.14	0.691
			HT	9	0.10	0.799
			T	44	–0.07	0.658
IVF	Basal pHi (F/Fmax)	Fertilization rate (%)	AT	3	–0.98	0.115
			HT	10	–0.37	0.288
			T	83	0.13	0.237
	Delta pHi		AT	3	0.95	0.209
			HT	10	0.33	0.35
			T	83	–0.07	0.508
	Semen volume (mL)		AT	3	0.35	0.775
			HT	10	0.17	0.633
			T	82	–0.03	0.778
	Concentration (cells × 10^6)		AT	3	–1.00	**0.025**
			HT	10	0.14	0.69
			T	83	–0.11	0.319
	Morphology (%)		AT	3	0.97	0.149
			HT	10	0.09	0.809
			T	83	0.08	0.46
	Motility (%)		AT	3	–0.33	0.783
			HT	10	–0.50	0.142
			T	81	0.09	0.425

The values in bold indicate statistical significance

## Discussion

Despite some advances, the field of male infertility remains limited by an incomplete understanding of many causal factors and the underlying mechanisms of its etiology. To enhance our knowledge and improve treatment options, it is crucial to identify factors influencing male fertility, explore regulatory pathways, and discover diagnostic biomarkers. This will, in turn, enable the development of therapeutic tools to assist patients in their fertility treatments [22]. As part of addressing this challenge, certain research efforts have focused on physiological parameters, such as measuring the resting membrane potential of the sperm (Em) [23–25], analyzing progesterone-induced increases in Ca^2^⁺ [25, 26], and the alkalinization of sperm pHᵢ [11, 25], as indicators of human sperm fertilizing capacity.

From spermatogenesis in the testes to fertilization in the female reproductive tract, a carefully controlled pH environment is essential for optimal sperm performance. Various regions, such as the testes, epididymis, and seminal fluid, maintain distinct pH levels that are precisely regulated to support specific sperm functions [4]. During capacitation, sperm pHᵢ increases as they transit through the female reproductive tract [9]. This alkalinization is essential for activating sperm motility and promoting the acrosome reaction [9, 27–30]. The pH in the female reproductive tract forms a gradient, starting with an acidic environment in the vagina (pH≈4.3) and gradually increasing through the cervix (pH≈6.5–7.5) to the fallopian tubes (pH≈8) [31]. This unique pH landscape may play a role in sperm selection and fertilization [4, 31]. Human spermatozoa possess intricate mechanisms to regulate pHᵢ. Key players include transporters, ion channels, and enzymes, such as Na⁺/H⁺ exchangers (NHEs), Cl⁻/HCO₃⁻ exchangers, and carbonic anhydrases (CAs), which mediate the exchange of H⁺ ions with other ions. Additionally, voltage-gated proton channels (Hv1) and K⁺ channels (SLO3/SLO1) contribute to pHᵢ regulation [6, 10, 32, 33]. The intracellular signaling pathways involving cAMP, cGMP, and Ca^2^⁺ also influence pHᵢ homeostasis [34–36]. Cytoplasmic alkalinization of sperm during capacitation also regulates the opening of the flagellar-specific Ca^2^⁺ channel CatSper, the main channel that regulates Ca^2^⁺ entry into sperm. The opening of CatSper induces the elevation of intracellular Ca^2^⁺ levels that are essential to facilitate various sperm processes, including hyperactivation, capacitation, and the acrosome reaction [10, 37]. All these signaling mechanisms are essential for sperm to acquire the ability to fertilize the oocyte.

Dysregulation of pHᵢ can trigger a cascade of adverse effects on sperm function. The inability to properly regulate pHᵢ can impair sperm motility, capacitation, and the acrosome reaction [10]. The acrosome and its remodeling following the AR play a fundamental role in successful fertilization [38, 39], as low fertilization rates have been observed when intracytoplasmic sperm injection (ICSI) is performed using sperm with intact acrosomes [40] or round sperm that lack this structure [41]. These findings suggest that alkalization of human sperm is important to help enhance fertilization rates during IVF and ICSI, by regulating the RA process and potentially improving pronuclear fusion. In patients with teratozoospermia, various abnormalities in the plasma membrane and acrosome (difficult to diagnose) can impair fertilization, even when sperm are directly injected into the oocyte cytoplasm. This can explain at least in part the correlation observed in this work between the increase in sperm pHᵢ with the increase in fertilization rates by ICSI.

Moreover, changes in the surrounding pH can increase the susceptibility of spermatozoa to oxidative stress and DNA damage [42]. These impairments can ultimately result in infertility or reduced fertility in affected individuals, as well as suboptimal outcomes in ART [11]. In our results, the basal sperm pHᵢ from non-normozoospermic patients undergoing ART treatments exhibited more alkaline basal pHᵢ levels compared to normozoospermic men; however, their values were not statistically significant. These differences are attributed to the small number of normozoospermic men who participated in this study and the fact that they did not undergo fertility treatment. This likely contributed to the high heterogeneity in data, which is a limitation of this study.

In this work, we evaluated the relevance of sperm pHᵢ in fertilization success in patients undergoing ART treatments, including both conventional IVF and ICSI cycles. We found a significant positive correlation between basal sperm pHᵢ and fertilization rates for only teratozoospermic (isolates) samples undergoing ICSI treatment, indicating that the more alkaline the basal pHᵢ of the sperm, the higher the likelihood of fertilization success. This correlation was not observed when conventional IVF was used. In contrast, Gunderson et al. (2021) reported a positive correlation between sperm pHᵢ and increased fertilization rates in normozoospermic patients undergoing conventional IVF. They suggested that sperm with higher pHᵢ values tend to have higher fertilization potential, likely due to enhanced motility and acrosome reaction capabilities [11]. The differences in our findings may be attributed to the fact that our study population consisted of non-normozoospermic patients diagnosed with teratozoospermia (characterized by abnormal sperm morphology), whereas Gunderson’s study focused on normozoospermic patients. Other important differences include the time and culture media used. In this research, we performed the qualitative measurement of pHᵢ around 2 to 3 h after sperm recovery in HTF medium supplemented with 10% HSA. In contrast, Gunderson et al. incubated sperm in Quinn’s Advantage capacitating media for around 18 h. They stained the cells with the pHᵢ-sensitive dye, centrifuged the sperm, and resuspended them in non-capacitating human tubal fluid (HTF) medium with 25 mM NaHCO₃^−^. The incubation conditions can influence the oocytes and embryonic development; therefore, it is important to consider that the studies differ in terms of the methodology used to evaluate pHᵢ. The differences in methodology and media used must be considered, as they can influence the physiological responses of the gametes. This highlights the need to develop more standardized protocols to evaluate sperm pH_i_.

In that sense, maintaining an optimal pH in the culture medium is essential for the survival and function of both sperm and oocytes. After cumulus cell removal during ICSI, oocytes are more vulnerable to pH fluctuations and rely heavily on the surrounding buffer [43]. In a recent article, Mendola and colleagues (2024) showed that the influx of zwitterionic buffers such as HEPES, bicarbonate, and MOPS employed after ICSI can inhibit various cellular processes, including the activity of protein transporters, ion channels, and mitochondrial functions. This was tested in human oocytes (MII stage) to evaluate the influence of buffer entry on the oocyte transcriptome after membrane perforation. These buffers can also interact with DNA, lipids, and metal ions, potentially altering oocyte development. As a result, the authors recommend using a bicarbonate buffer for oocyte retention during ICSI [44]. This finding is significant as it highlights that those ions and the pH of the surrounding environment, and possibly the sperm pH_i_, could affect oocyte activation and embryonic development. More studies are needed to explore the relationship between sperm, oocyte activation, and embryonic development. On the other hand, it is necessary to continue studying and thoroughly evaluate the relevance of culture media on the physiology and function of gametes and embryos during ART application.

Additionally, the regulation of pH is essential during zygote formation, as it also impacts critical processes such as oocyte activation, pronucleus formation, and early embryonic development. Proper pH levels support mitochondrial function, enzyme activation, and energy production, all of which are required for the successful transition from fertilization to the first stages of embryogenesis. In ART procedures, maintaining an optimal pH environment is equally vital to ensure fertilization success and zygote viability. Although the exact role of sperm pH_i_ in fertilization remains unclear, these results suggest that sperm pH_i_ may play a crucial role in regulating not only the fusion process between sperm and egg but also the subsequent steps to obtain a healthy embryo.

While semen analysis provides valuable information about sperm quality, it does not directly evaluate a sperm’s ability to fertilize an egg as shown in this study where no correlation was found between semen macroscopic parameters and fertilization rate. Therefore, it is necessary to search for diagnostic tools with greater precision that improve the probability of success during IVF procedures. Alternatives, such as measuring pH_i_ in capacitated sperm, could be a promising tool to help clinicians choose the best ART for each patient. The protocol presented here is quick and easy to perform in a laboratory that has a flow cytometer. One of the advantages of this qualitative method is that it does not require carrying out pH_i_ calibration curves, which can be considerably more complex and may require media with different pHs. Furthermore, this indirect method of assessing basal pH_i_ (basal and delta pH_i_), a fundamental parameter in the molecular processes involved in sperm function, offers the potential to provide valuable, more specific information on disturbances in male fertility.

## Data Availability

The data supporting the findings of this study are available in the tables uploaded in this submission.

## References

[1] Cox CM, Thoma ME, Tchangalova N, Mburu G, Bornstein MJ, Johnson CL, Kiarie J. Infertility prevalence and the methods of estimation from 1990 to 2021: a systematic review and meta-analysis. Hum Reprod Open. 2022;2022(4):hoac051. 10.1093/hropen/hoac051. 36483694 10.1093/hropen/hoac051 PMC9725182

[2] World Health Organization. Laboratory manual for the examination and processing of human semen. 6th ed. Geneva; 2021.

[3] Nishigaki T, José O, González-Cota AL, Romero F, Treviño CL, Darszon A. Intracellular pH in sperm physiology. Biochem Biophys Res Commun. 2014;450:1149–58. 10.1016/j.bbrc.2014.05.100. 24887564 10.1016/j.bbrc.2014.05.100 PMC4146485

[4] Dai P, Zou M, Cai Z, Zeng X, Zhang X, Liang M, et al. pH homeodynamics and male fertility: a coordinated regulation of acid-based balance during sperm journey to fertilization. Biomolecules. 2024;14:685. 10.3390/biom14060685. 38927088 10.3390/biom14060685 PMC11201807

[5] Ren D, Navarro B, Perez G, Jackson AC, Hsu S, Shi Q, et al. A sperm ion channel required for sperm motility and male fertility. Nature. 2001;413:603–9. 10.1038/35098027. 10.1038/35098027 PMC8462998 11595941

[6] Lishko PV, Kirichok Y. The role of Hv1 and CatSper channels in sperm activation. J Physiol. 2010;588:4667–72. 10.1113/jphysiol.2010.194142. 20679352 10.1113/jphysiol.2010.194142 PMC3010136

[7] Chávez JC, De La Vega-Beltrán JL, Escoffier J, Visconti PE, Treviño CL, Darszon A, et al. Ion permeabilities in mouse sperm reveal an external trigger for SLO3-dependent hyperpolarization. Travis AJ, editor. PLoS ONE. 2013;8:e60578. 10.1371/journal.pone.0060578. 10.1371/journal.pone.0060578 PMC3618424 23577126

[8] Delgado-Bermúdez A, Yeste M, Bonet S, Pinart E. A review on the role of bicarbonate and proton transporters during sperm capacitation in mammals. Int J Mol Sci. 2022;23:6333. 10.3390/ijms23116333. 35683013 10.3390/ijms23116333 PMC9180951

[9] Matamoros-Volante A, Treviño CL. Capacitation-associated alkalization in human sperm is differentially controlled at the subcellular level. J Cell Sci. 2020;133:jcs238816. 10.1242/jcs.238816. 31932506 10.1242/jcs.238816

[10] Chávez JC, Carrasquel-Martínez G, Hernández-Garduño S, Matamoros Volante A, Treviño CL, Nishigaki T, et al. Cytosolic and acrosomal pH regulation in mammalian sperm. Cells. 2024;13:865. 10.3390/cells13100865. 38786087 10.3390/cells13100865 PMC11120249

[11] Gunderson SJ, Puga Molina LC, Spies N, Balestrini PA, Buffone MG, Jungheim ES, et al. Machine-learning algorithm incorporating capacitated sperm intracellular pH predicts conventional in vitro fertilization success in normospermic patients. Fertil Steril. 2021;115:930–9. 10.1016/j.fertnstert.2020.10.038. 10.1016/j.fertnstert.2020.10.038 PMC9110269 33461755

[12] Agarwal A, Tvrda E, Sharma R, Springer Science and Business Media LLC. Relationship amongst teratozoospermia, seminal oxidative stress and male infertility. Reprod Biol Endocrinol. 2014;12:45. 10.1186/1477-7827-12-45. 24884815 10.1186/1477-7827-12-45 PMC4049374

[13] Ammar O, Mehdi M, Muratori M. Teratozoospermia: its association with sperm DNA defects, apoptotic alterations, and oxidative stress. Andrology. 2020;8:1095–106. 10.1111/andr.12778. 32096605 10.1111/andr.12778

[14] Atmoko W, Savira M, Shah R, Chung E, Agarwal A, AME Publishing Company. Isolated teratozoospermia: revisiting its relevance in male infertility: a narrative review. Transl Androl Urol. 2024;13:260–73. 10.21037/tau-23-397. 38481866 10.21037/tau-23-397 PMC10932644

[15] Hotaling JM, Smith JF, Rosen M, Muller CH, Walsh TJ, Elsevier BV. The relationship between isolated teratozoospermia and clinical pregnancy after in vitro fertilization with or without intracytoplasmic sperm injection: a systematic review and meta-analysis. Fertil Steril. 2011;95:1141–5. 10.1016/j.fertnstert.2010.09.029. 21030014 10.1016/j.fertnstert.2010.09.029

[16] El Khattabi L, Dupont C, Sermondade N, Hugues J-N, Poncelet C, Porcher R, et al. Is intracytoplasmic morphologically selected sperm injection effective in patients with infertility related to teratozoospermia or repeated implantation failure? Fertil Steril. 2013;100:62–8. 10.1016/j.fertnstert.2013.02.048. 10.1016/j.fertnstert.2013.02.048 23548938

[17] Candela L, Boeri L, Capogrosso P, Cazzaniga W, Pozzi E, Belladelli F, et al. Correlation among isolated teratozoospermia, sperm DNA fragmentation and markers of systemic inflammation in primary infertile men. Schlatt S, editor. PLOS ONE. Publ Libr Sci (PLoS); 2021;16:e0251608. 10.1371/journal.pone.0251608. 10.1371/journal.pone.0251608 PMC8184012 34097690

[18] Zheng D, Zeng L, Yang R, Lian Y, Zhu Y-M, Liang X, et al. Intracytoplasmic sperm injection (ICSI) versus conventional in vitro fertilisation (IVF) in couples with non-severe male infertility (NSMI-ICSI): protocol for a multicentre randomised controlled trial. BMJ Open. 2019;9:e030366. 10.1136/bmjopen-2019-030366. 10.1136/bmjopen-2019-030366 PMC6773417 31575574

[19] Alpha Scientists in Reproductive Medicine and ESHRE Special Interest Group of Embryology, Balaban B, Brison D, Calderon G, Catt J, Conaghan J, et al. The Istanbul consensus workshop on embryo assessment: proceedings of an expert meeting. Hum Reprod. 2011;26:1270–83. 10.1093/humrep/der037. 10.1093/humrep/der037 21502182

[20] R Core Team. R: A Language and Environment for Statistical Computing. Vienna, Austria; 2017. https://www.R-project.org/

[21] Gómez-Rubio V. ggplot2 - elegant graphics for data analysis (2nd edition). J Stat Softw [Internet]. Foundation for Open Access Statistic; 2017 [cited 2025 July 13];77. 10.18637/jss.v077.b02.

[22] Wang F, Yang W, Ouyang S, Yuan S, MDPI AG. The vehicle determines the destination: the significance of seminal plasma factors for male fertility. Int J Mol Sci. 2020;21:8499. 10.3390/ijms21228499. 33198061 10.3390/ijms21228499 PMC7696680

[23] Baro Graf C, Ritagliati C, Torres-Monserrat V, Stival C, Carizza C, Buffone MG, et al. Membrane potential assessment by fluorimetry as a predictor tool of human sperm fertilizing capacity. Front Cell Dev Biol. 2020;7:383. 10.3389/fcell.2019.00383. 10.3389/fcell.2019.00383 PMC6979052 32010695

[24] Molina LCP, Gunderson S, Riley J, Lybaert P, Borrego-Alvarez A, Jungheim ES, et al. Membrane potential determined by flow cytometry predicts fertilizing ability of human sperm. Front Cell Dev Biol. 2020;7:387. 10.3389/fcell.2019.00387. 10.3389/fcell.2019.00387 PMC6985285 32039203

[25] Matamoros-Volante A, Castillo-Viveros V, Torres-Rodríguez P, Treviño MB, Treviño CL. Time-lapse flow cytometry: a robust tool to assess physiological parameters related to the fertilizing capability of human sperm. Int J Mol Sci. 2020;22:93. 10.3390/ijms22010093. 33374265 10.3390/ijms22010093 PMC7796328

[26] Kelly MC, Brown SG, Costello SM, Ramalingam M, Drew E, Publicover SJ, et al. Single-cell analysis of [Ca2+]i signalling in sub-fertile men: characteristics and relation to fertilization outcome. Hum Reprod. 2018;33:1023–33. 10.1093/humrep/dey096. 10.1093/humrep/dey096 PMC5972555 29697805

[27] Pinto FM, Odriozola A, Candenas L, Subirán N, MDPI AG. The role of sperm membrane potential and ion channels in regulating sperm function. Int J Mol Sci. 2023;24:6995. 10.3390/ijms24086995. 37108159 10.3390/ijms24086995 PMC10138380

[28] Chávez JC, De La Vega-Beltrán JL, José O, Torres P, Nishigaki T, Treviño CL, et al. Acrosomal alkalization triggers Ca2+ release and acrosome reaction in mammalian spermatozoa. J Cell Physiol. 2018;233:4735–47. 10.1002/jcp.26262. 29135027 10.1002/jcp.26262

[29] Carrasquel Martínez G, Aldana A, Carneiro J, Treviño CL, Darszon A. Acrosomal alkalinization occurs during human sperm capacitation. Mol Hum Reprod. 2022;28:gaac005. 10.1093/molehr/gaac005. 35201340 10.1093/molehr/gaac005

[30] Ferreira JJ, Lybaert P, Puga-Molina LC, Santi CM. Conserved mechanism of bicarbonate-induced sensitization of CatSper channels in human and mouse sperm. Front Cell Dev Biol [Internet]. Frontiers Media SA; 2021 [cited 2025 July 13];9. 10.3389/fcell.2021.733653. 10.3389/fcell.2021.733653 PMC8505895 34650979

[31] Ng KYB, Mingels R, Morgan H, Macklon N, Cheong Y, Oxford University Press (OUP). In vivo oxygen, temperature and pH dynamics in the female reproductive tract and their importance in human conception: a systematic review. Hum Reprod Update. 2018;24:15–34. 10.1093/humupd/dmx028. 29077897 10.1093/humupd/dmx028

[32] Santi CM, Martínez-López P, De La Vega-Beltrán JL, Butler A, Alisio A, Darszon A, et al. The SLO3 sperm-specific potassium channel plays a vital role in male fertility. FEBS Lett. 2010;584:1041–6. 10.1016/j.febslet.2010.02.005. 10.1016/j.febslet.2010.02.005 PMC2875124 20138882

[33] Berger TK, Fußhöller DM, Goodwin N, Bönigk W, Müller A, Dokani Khesroshahi N, et al. Post-translational cleavage of Hv1 in human sperm tunes pH- and voltage-dependent gating. J Physiol. 2017;595:1533–46. 10.1113/jp273189. 27859356 10.1113/JP273189 PMC5330862

[34] Carlson AE, Hille B, Babcock DF, Elsevier BV. External Ca2+ acts upstream of adenylyl cyclase SACY in the bicarbonate signaled activation of sperm motility. Dev Biol. 2007;312:183–92. 10.1016/j.ydbio.2007.09.017. 17950270 10.1016/j.ydbio.2007.09.017 PMC2259292

[35] Buffone MG, Wertheimer EV, Visconti PE, Krapf D, Elsevier BV. Central role of soluble adenylyl cyclase and cAMP in sperm physiology. Biochimica et Biophysica Acta (BBA). 2014;1842:2610–20. 10.1016/j.bbadis.2014.07.013. 25066614 10.1016/j.bbadis.2014.07.013 PMC4262597

[36] Puga Molina LC, Pinto NA, Torres Rodríguez P, Romarowski A, Vicens Sanchez A, Visconti PE, et al. Essential role of CFTR in PKA-dependent phosphorylation, alkalinization, and hyperpolarization during human sperm capacitation. J Cell Physiol. 2017;232:1404–14. 10.1002/jcp.25634. 27714810 10.1002/jcp.25634 PMC5548537

[37] Hwang JY, Chung J-J, American Physiological Society. CatSper calcium channels: 20 years on. Physiology. 2023;38:125–40. 10.1152/physiol.00028.2022. 10.1152/physiol.00028.2022 PMC10085559 36512352

[38] Siu KK, Serrão VHB, Ziyyat A, Lee JE. The cell biology of fertilization: gamete attachment and fusion. J Cell Biol. 2021;220:e202102146. 10.1083/jcb.202102146. 34459848 10.1083/jcb.202102146 PMC8406655

[39] Okabe M. The acrosome reaction: a historical perspective. In: Buffone MG, editor. Sperm acrosome Biog Funct Fertil [Internet]. Cham: Springer International Publishing; 2016 [cited 2025 Sept 24]. p. 1–13. 10.1007/978-3-319-30567-7_1.

[40] Morozumi K, Yanagimachi R. Incorporation of the acrosome into the oocyte during intracytoplasmic sperm injection could be potentially hazardous to embryo development. Proc Natl Acad Sci U S A. 2005;102:14209–14. 10.1073/pnas.0507005102. 16183738 10.1073/pnas.0507005102 PMC1242329

[41] Dávila Garza SA, Patrizio P. Reproductive outcomes in patients with male infertility because of Klinefelter’s syndrome, Kartagener’s syndrome, round-head sperm, dysplasia fibrous sheath, and ‘stump’ tail sperm: an updated literature review. Curr Opin Obstet Gynecol. 2013;25:229–46. 10.1097/GCO.0b013e32835faae5. 23587797 10.1097/GCO.0b013e32835faae5

[42] Castro JS, Braz-Mota S, Campos DF, Souza SS, Val AL, Frontiers Media SA. High temperature, pH, and hypoxia cause oxidative stress and impair the spermatic performance of the Amazon fish *Colossoma macropomum*. Front Physiol. 2020. 10.3389/fphys.2020.00772. 32733277 10.3389/fphys.2020.00772 PMC7360832

[43] FitzHarris G, Baltz JM, Bioscientifica. Regulation of intracellular pH during oocyte growth and maturation in mammals. Reproduction. 2009;138:619–27. 10.1530/rep-09-0112. 19520797 10.1530/REP-09-0112

[44] Mendola RJ, Biswas L, Schindler K, Walmsley RH, Russell H, Angle M, et al. Influx of zwitterionic buffer after intracytoplasmic sperm injection (ICSI) membrane piercing alters the transcriptome of human oocytes. J Assist Reprod Genet. 2024;41:1341–56. 10.1007/s10815-024-03064-2. 10.1007/s10815-024-03064-2 PMC11143126 38436798

